# Decision-making in Multiple Sclerosis: The Role of Aversion to Ambiguity for Therapeutic Inertia among Neurologists (DIScUTIR MS)

**DOI:** 10.3389/fneur.2017.00065

**Published:** 2017-03-01

**Authors:** Gustavo Saposnik, Angel P. Sempere, Daniel Prefasi, Daniel Selchen, Christian C. Ruff, Jorge Maurino, Philippe N. Tobler

**Affiliations:** ^1^Division of Neurology, Stroke Outcomes and Decision Neuroscience Research Unit, Department of Medicine, St. Michael’s Hospital, University of Toronto, Toronto, ON, Canada; ^2^Laboratory for Social and Neural Systems Research, Department of Economics, University of Zurich, Zurich, Switzerland; ^3^Li Ka Shing Knowledge Institute, St. Michael’s Hospital, University of Toronto, Toronto, ON, Canada; ^4^Department of Neurology, Hospital General Universitario de Alicante, Alicante, Spain; ^5^Neuroscience Area, Medical Department, Roche Farma, Madrid, Spain

**Keywords:** multiple sclerosis, disease-modifying therapy, neuroeconomics, decision-making, risk aversion

## Abstract

**Objectives:**

Limited information is available on physician-related factors influencing therapeutic inertia (TI) in multiple sclerosis (MS). Our aim was to evaluate whether physicians’ risk preferences are associated with TI in MS care, by applying concepts from behavioral economics.

**Design:**

In this cross-sectional study, participants answered questions regarding the management of 20 MS case scenarios, completed 3 surveys, and 4 experimental paradigms based on behavioral economics. Surveys and experiments included standardized measures of aversion ambiguity in financial and health domains, physicians’ reactions to uncertainty in patient care, and questions related to risk preferences in different domains. The primary outcome was TI when physicians faced a need for escalating therapy based on clinical (new relapse) and magnetic resonance imaging activity while patients were on a disease-modifying agent.

**Results:**

Of 161 neurologists who were invited to participate in the project, 136 cooperated with the study (cooperation rate 84.5%) and 96 completed the survey (response rate: 60%). TI was present in 68.8% of participants. Similar results were observed for definitions of TI based on modified Rio or clinical progression. Aversion to ambiguity was associated with higher prevalence of TI (86.4% with high aversion to ambiguity vs. 63.5% with lower or no aversion to ambiguity; *p* = 0.042). In multivariate analyses, high aversion to ambiguity was the strongest predictor of TI (OR 7.39; 95%CI 1.40–38.9), followed by low tolerance to uncertainty (OR 3.47; 95%CI 1.18–10.2).

**Conclusion:**

TI is a common phenomenon affecting nearly 7 out of 10 physicians caring for MS patients. Higher prevalence of TI was associated with physician’s strong aversion to ambiguity and low tolerance of uncertainty.

## Introduction

Making decisions in medical care is a complex task (1). Physicians have limited education in both risk management and decision-making at medical schools (2). Decisions based on erroneous assessments may result in incorrect patient and family expectations, and potentially suboptimal advice, treatment, and outcome.

In behavioral economics, *uncertainty* is a generic term that comprises risk and ambiguity. *Risk* applies to events with known probability (3). In contrast, *ambiguity* is a term reserved for events for which probabilities are unknown (3). Typically, people are averse to both ambiguity and risk, and the two aversions are independent of each other (4). Uncertainty is one of the most important contributing factors affecting decisions in medical care (5, 6). However, limited information is available regarding the role of aversion to ambiguity in medical decisions.

Appropriate multiple sclerosis (MS) care involves complex medical decisions as it requires consideration of multiple short- and long-term factors (e.g., imaging results, disease progression, patient’s characteristics, and their preferences, etc.). No evidence of disease activity is emerging as a new standard for treatment response and may be associated with improved long-term disability outcomes. A more proactive management strategy, including earlier use of high-efficacy DMTs and close monitoring of the clinical and radiological response to treatment, is recommended to slow the progression of physical and cognitive impairments in patients with relapsing-remitting multiple sclerosis (RRMS) (7–9). Treatment escalation has been shown to reduce relapse rates, disability progression, and magnetic resonance imaging (MRI) activity (10).

Therapeutic inertia (TI) is a term introduced in 2006 to define the absence of treatment initiation or intensification in patients when treatment goals are unmet (11–14). In the context of MS, TI is defined as the lack of treatment initiation or escalation when there is evidence of disease activity (based on the clinical course and neuroimaging markers) (15, 16). It is possible that aversion to ambiguity contributes to TI as the probabilities of benefits with treatment escalation are typically less well known than with treatment continuation. To address this possibility, we need a better understanding of physician-related factors influencing decisions about DMTs and the prevalence of TI in MS care. The application of experiments from behavioral economics would facilitate the recognition of physicians’ therapeutic preferences and beliefs about DMTs for MS in the real world (17).

We hypothesized that physicians’ ambiguity aversion or low tolerance to uncertainty are associated with TI and clinical decisions in MS care. In the present study, we thus assessed the prevalence of TI (and associated contributing factors) in typical clinical decisions among physicians caring for MS patients across Spain.

## Materials and Methods

**Figure 1:**
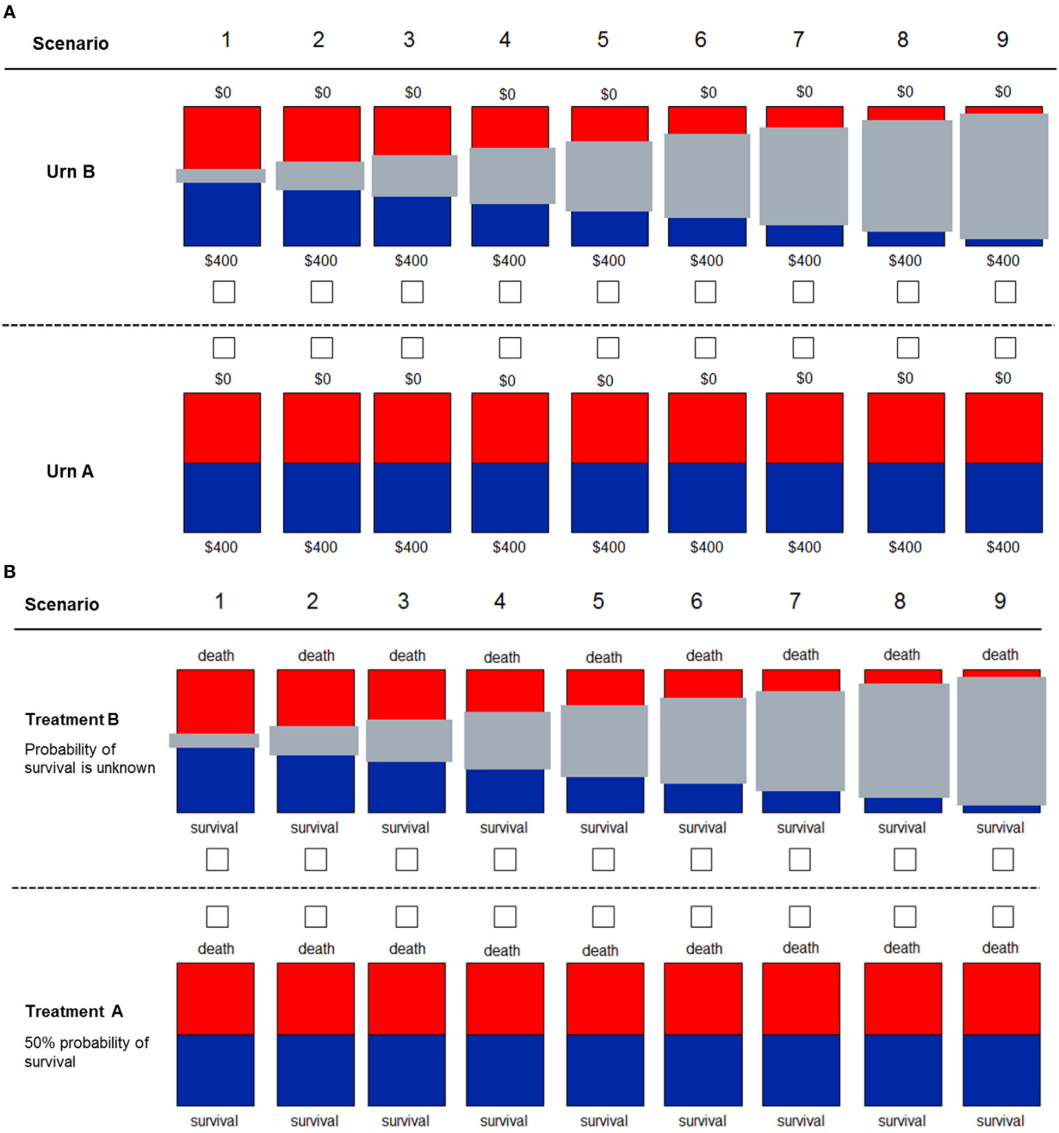
**Decision scenarios used to measure ambiguity in financial (A) and health (B) domains**. Participants were told to imagine two different types or urns. For urn type A, they knew that 50% of the balls were red and the other 50% were blue. For urn type B, they did not know the exact proportion of blue to red balls, with the gray bar representing the unknown proportion of balls. For the financial domain, participants knew that if they drew a blue ball, they would win the full amount of $400. If they drew a red ball, they would win $0. For the health domain, participants decided between two treatments for a patient. With “Treatment A,” the patient had a 50% probability of survival. With “Treatment B,” the exact probability of survival was unknown, with the gray bar representing the unknown probability.

We conducted a web-based study using the Qualtrics platform (http://qualtrics.com). The study comprised 20 MS case-vignettes, 3 standardized surveys, and 4 behavioral experiments among practicing neurologists from Spain from November 3, 2015 to March 31, 2016 (see protocol published elsewhere) (15). In brief, participants answered three components in the following order: (i) demographic information, (ii) behavioral experiments/surveys, and (iii) case scenarios. Responses from case scenarios were analyzed in light of responses from the behavioral component. MS case scenarios were derived from the most common situations in clinical practice as identified by experts in the field (Drs. Daniel Selchen and Angel P. Sempere). Behavioral experiments were designed to assess risk and ambiguity aversion in the health and financial domains (exposure) (Figure 1) (15, 18, 19). Ambiguity aversion is defined as dislike for events with unknown probability over events with known probability (18). For example, an ambiguity-averse individual would rather choose a treatment where the probability of benefits or side effects are known (even if these are somewhat unfavorable) over one where these probabilities are unknown. Specifically, participants were asked to choose between a visual option with known 50/50 probability of winning €400 or €0 and an option with unknown probability of the same outcomes. Gray bars represented the degree to which the winning probability was unknown (Figure 1). The degree of ambiguity aversion was defined as the proportion of times participants chose the 50/50 option over the ambiguous option. As the overall level of ambiguity aversion was pronounced in our sample (mean 61.7% preference for 50/50 option, i.e., the option with known probabilities) and to avoid using an arbitrary criterion, we classified participants as highly ambiguity averse if they chose the 50/50 (known probability) option in each of the nine scenarios (Figure 1) (20). In order to evaluate the consistency of the relationship with the primary outcome, we also analyzed another definition of ambiguity aversion (choice of the known probability option instead of the option with the 50% unknown probability in scenario 5; Figure 1).

In principle, risk aversion is another factor that may influence clinical decisions (21). Risk aversion is defined as the tendency to prefer safe payoffs over probabilistic payoffs when the expected value of both options is identical (4, 18). A risk-averse patient would thus prefer a treatment that provides a small improvement with certainty over a treatment that provides a larger or no improvement with equal chance (50/50). We evaluated risk aversion by identifying the safe amount for which a participant was indifferent between the safe and the risky option (22). This indifference amount, called certainty equivalent, reflects the value associated to the risky option and facilitates comparison between participants. For example, participants were asked what would be the minimal certain payoff that they would prefer over the equiprobable gamble of winning €400 or €0 (expected value of €200). The degree of risk aversion of each individual corresponded to the difference of the expected value of the risky option (€200) minus the participant’s response (proxy of certainty equivalent). A similar visual design and methodology was used to elicit aversion to risk and ambiguity in the health domains (questions #15 and #17) (15). Participants were asked to choose between Treatment A (50% probability of survival) and “Treatment B” (the probability of survival is unknown) with the gray bars quantifying how much is unknown about the probability of survival.

We also used two standardized surveys to assess physicians’ willingness to take risks and tolerance to uncertainty. The German Socio-Economic Panel (SOEP) is a validated survey that evaluates willingness to take risks in different domains (financial matters, own health, driving, own occupation, etc.) (23). We used questions of the form: “How would you rate your willingness to take risks in the following areas….”? Areas included financial matters, driving, occupation, etc., and responses could range from 0 (not at all) to 10 (very much).

The second survey measured physicians’ tolerance to uncertainty in patient care, using the reaction to uncertainty test (24). It comprises five questions to be rated from 0 to 5 that when added gives a total score (25). Low tolerance to uncertainty was defined as values below the median of the total score. Further details of the protocol were published elsewhere (15).

### Participants

Practicing neurologists actively involved in the care of patients with MS from across Spain were invited to participate in our study by the Spanish Society of Neurology (Sociedad Española d8e Neurologia-SEN). Physicians whose practice was primarily in caring for MS patients were classified as “MS specialists.” All participants received compensation for completing the survey.

### Definitions

For the primary analysis, disease activity was defined as a clinical relapse plus the presence of new brain lesions in follow-up MRI scans with at least one gadolinium-enhancing lesion (26, 27). In a sensitivity analysis, we also used the European Medicines Agency (EMA) and the modified Rio criteria to evaluate variations in TI. For example, the EMA approves escalating therapy from interferon-beta to natalizumab or fingolimod in patients who had at least one relapse in the previous year and either ≥9 T2 hyperintense lesions or ≥1 gadolinium-enhancing T1 lesion on brain MRI (26, 27). The high-risk profile according to the modified Rio score includes either an MRI with more than 5 new T2 lesions (1 point) or 1 relapse in the first year (1 point) or two relapses within the first year of treatment (2 points) or the combination of these criteria (8, 28). The use of these definitions combining a clinical relapse and MRI activity is consistent with recent evidence regarding the risk of treatment failure among patients receiving interferon-beta (29).

Disease progression was defined as at least one point worsening from baseline in the Expanded Disability Status Scale (EDSS) score (Table S1 in Supplementary Material) (28).

Recent meta-analysis confirmed that alemtuzumab, natalizumab, and fingolimod are the best available choices for preventing clinical relapses in patients with RRMS (30). However, there is no consensus algorithm available despite the publication of national or regional recommendations (8, 16, 26, 31–33). As a result, the current landscape of DMTs for the treatment of RRMS includes first-line therapies (beta interferons, glatiramer acetate, teriflunomide, and dimethyl fumarate) and second-line therapies (fingolimod, natalizumab, and alemtuzumab). For the present analysis, we used the aforementioned scheme according to the current clinical practice.

### Outcome Measures

The primary outcome of the study was the proportion of participants who exhibited TI and its association with aversion to ambiguity (14, 18). TI (presence/absence) was determined as the lack of escalation of therapy given disease activity while patients received a DMT in at least one case scenario.

Secondary outcome measures included the association between tolerance to uncertainty, risk aversion, and the SOEP surveys, on the one hand, and with TI and therapeutic decisions, on the other hand.

### Statistical Analysis

The primary analysis assessed the possible association between physicians’ aversion to ambiguity and TI. A multiple logistic regression analysis with backward selection was completed to determine the association between physicians’ characteristics with the primary outcome of interest. We included the following explanatory variables: age, gender, MS patients seen per week, practice setting (academic vs. non-academic), % of time devoted to clinical care, coauthor in a peer-reviewed publication within the last 3 years (yes/no), attendance to the European Committee for Treatment and Research in Multiple Sclerosis 2015 annual meeting, risk aversion, overconfidence, tolerance to uncertainty (above/below the median), willingness to take risks in all domains (SOEP survey—above/below the median), and herding (following recommendations made by another colleague). As there was a high correlation between MS specialists (self-defined) and number of MS patients assessed per week (Spearman’s rho = 0.58; *p* < 0.001), only the latter was entered in the multivariate analysis. Linear regression analysis was used to test for a relation between the number of patients assessed per week and the outcomes of interest. A sensitivity analysis was completed by using different criteria of TI and building models including all variables of interest (Supplementary Material).

All tests were 2-tailed, and *p*-values <0.05 were considered significant. We calculated the power of the study for the primary outcome of interest with an alpha error level of 0.05 and found that we had 100% power to detect a 27% difference between groups for the primary outcome measure.

The study was approved by the Research Ethics Board of St. Michael’s Hospital, University of Toronto, ON, Canada.

## Results

Out of the 161 neurologists who were invited to participate in the study from representative areas of Spain, 136 cooperated (cooperation rate 84.5%) and 96 completed the survey (response rate 60%). There was representation from all regional territories except the Canary Islands (Figure S1 in Supplementary Material).

Overall, the mean (SD) age was 39.5 (±8.5) years; 51 (53%) were female. Two-thirds primarily focused their practice on MS care (*n* = 64; 66.7%). The mean years in practice was 14, commonly assessing 20 (±15) MS patients per week. Table 1 summarizes baseline characteristics of the study population.

**Table 1:** **Baseline characteristics of participants**.

Characteristics	
**Age** (mean ± SD), in years	39.5 ± 8.5
**Sex**	**No. of participants (%)**
Male	45 (46.9)
Female	51 (53.1)
**Specialty**	
Multiple sclerosis (MS) specialist	64 (66.7)
General neurologist who care for MS patients	32 (33.3)
**Practice setting**	
Academic	48 (50.0)
Community	26 (27.1)
Both (academic and community)	21 (21.9)
Other	1 (1.0)
**% time in clinical practice**	
>75%	70 (72.9)
**Years in practice**, mean ± SD	14.1 ± 10
**MS patients seen per week**, mean ± SD	20 ± 15
**Attended latest European Committee for Treatment and Research in Multiple Sclerosis conference**	56 (58)
**Author of a peer-reviewed publication in the last 3 years**	79 (82.3)

For the measurement of risk preferences, the mean safe payoffs were €200 (±33) in the financial domain and 12.3 (±4.3) years in the health domain.

For the measurement of ambiguity, total aversion to unknown probability (all nine scenarios) was observed in 23% of participants in the financial domain and 27.1% for the health domain. For the scenario where the ambiguous option contained 50% unknown probability (scenario 5), 59.4% of participants chose the known probability (50/50) option in the financial domain and 73.7% in the health domain. The median time for completing the study was 39 min (IQR 30–52 min).

Therapeutic inertia was present in 68.8% of participants. Similar findings were observed when we applied the modified Rio criteria (61.5%), modified Rio or neurological progression (67.7%), but TI was less common (29.2%) when we applied the EMA criteria. TI was less common among MS specialists (Table 2). Moreover, a higher number of MS patients seen per week were associated with a significantly lower risk of TI. Linear regression analysis suggests that the assessment of 10 more MS patients per week (from a baseline of 16) was associated with lower risk of TI (adjusted coefficient −10.2; 95%CI −18.4 to −2.0).

**Table 2:** **Prevalence of therapeutic inertia (TI) among multiple sclerosis (MS) specialists and general neurologists**.

Outcome	MS specialists *N* = 64 (66.7)	General neurologists *N* = 32 (33.3)	*p*-Value
**TI (criterion)**			
Clinico-radiological	40 (62.5)	26 (81.3)	0.062
European Medicines Agency	13 (20.3)	15 (46.9)	0.007
Modified Rio or progression	39 (60.9)	26 (81.3)	0.045

### Aversion to Ambiguity and TI

For the primary outcome, high aversion to ambiguity in the financial domain was associated with TI (86.4 vs. 63.5%; *p* = 0.042). High ambiguity aversion in the health domain was not associated with TI (76.9 vs. 65.7%; adjusted OR 1.79, 95%CI 0.61–5.25). Multivariable logistic regression analysis showed that high aversion to ambiguity in the financial domain was the strongest predictor of TI, significantly stronger even than aversion to ambiguity in the health domain (adjusted OR 7.39; 1.40–38.9) (Table 3; Table S2 and Figures S2 and S3 in Supplementary Material). The results were also consistent when ambiguity aversion was defined by 50% unknown probability (adjusted OR 3.29; 1.21–8.99).

**Table 3:** **Effect of high ambiguity aversion according to different definitions of therapeutic inertia (TI)**.

Outcome	Prevalence (%) of TI in the cohort	Adjusted model for ambiguity aversion^a^		Adjusted model for ambiguity aversion^b^	
		OR (95%CI)	*c*-Statistics	OR (95%CI)	*c*-Statistics
**TI (criterion)**					
Clinico-radiological	66 (68.8)	7.39 (1.40–38.9)	0.804	8.01 (1.01–73.3)	0.828
European Medicines Agency	28 (29.2)	8.02 (1.37–37.1)	0.777	7.17 (1.36–37.6)	0.796
Modified Rio or progression (Expanded Disability Status Scale >1)	65 (67.7)	4.41 (1.04–18.7)	0.791	4.01 (0.83–19.3)	0.811

*^a^Models derived from stepwise logistic regression with backward selection with p > 0.2 level for removal*.

*^b^Models derived from logistic regression including all variables of interest (age, sex, number of multiple sclerosis patients seen per week, practice setting, academic profile, risk aversion, ambiguity aversion, tolerance to uncertainty, herding, and overconfidence)*.

A sensitivity analysis showed that high aversion to ambiguity was also the strongest predictor of TI when applying the EMA criteria (adjusted OR 8.01; 95%CI 1.73–37.1) for the composite outcome of disease activity (modified Rio criteria) or evidence of progression (OR 4.41; 95%CI 1.04–18.7). The results remained consistent when models included all explanatory variables of interest, including number of patients seen per week (Table S2 in Supplementary Material).

**Figure 2:**
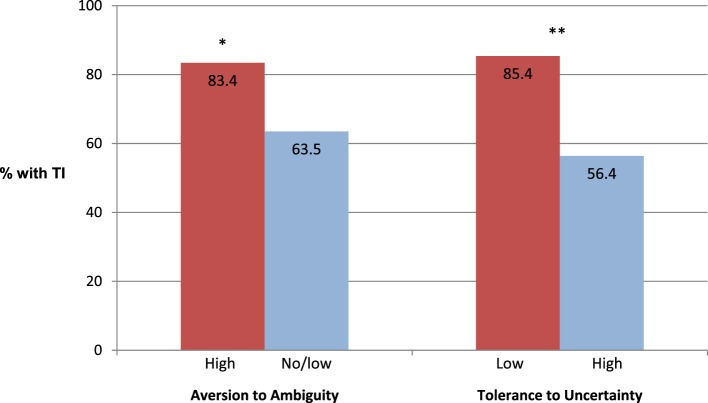
**Prevalence of therapeutic inertia (TI) among participants with high ambiguity aversion in the financial domain and low tolerance to uncertainty in patient care**. See description in the text for the criteria of TI. *p = 0.042; **p < 0.01.

Low tolerance to uncertainty (physician’s reaction to uncertainty survey) was associated with higher prevalence of TI (85.4 vs. 56.4%; adjusted OR 4.73, 1.63–13.7) (Figure 2). The association between TI and low tolerance to uncertainty was independent of the association between TI and high ambiguity aversion (Table S2 in Supplementary Material).

Conversely, willingness to take risk in multiple domains (as measured by the SOEP survey) or herding was not associated with TI (Table S2 in Supplementary Material).

## Discussion

Multiple sclerosis patients and their treating physicians are routinely confronted with uncertainties concerning the diagnosis, prognosis, disease course, and disease-modifying therapies (27).

In the present study, we applied validated experiments and surveys from behavioral economics to evaluate the association between physicians’ aversion to risk or ambiguity and TI (15). We found that TI affects nearly 7 out of 10 neurologists caring for MS patients but was less common among physicians with greater patient volumes per week or MS specialists. High aversion to ambiguity was the strongest predictor of TI even after adjusting for relevant confounders (e.g., age, practice setting, years in practice, percentage of time in clinical practice, overconfidence, time of survey completion, etc.). Lower tolerance to uncertainty was also associated with 3.5-fold higher risk of TI. On average, the assessment of 10 more MS patients per week was associated with lower risk of TI. Our results were consistent when employing various criteria on when to escalate therapy, as defined based on disease activity and/or progression. Physicians’ characteristics (e.g., age, gender, practice setting, years in practice, percentage of time in clinical practice) were not associated with TI.

In the last few years, there has been an increase in the availability and variability of therapeutic options for the management of MS. Although having more options is perceived as beneficial, consumer studies in psychology suggest the higher the number of options, the more difficult the decision, leading to a higher number of less satisfactory choices (labeled as “choice overload”) (34, 35).

Our results have practical implications that deserve comment. We showed that either a simple experiment or a short survey outside of the medical domain that assess aversion to ambiguity or tolerance to uncertainty may help to identify TI among neurologists and MS experts. The lack of escalation of therapies may lead to greater disability of MS patients, increasing the health-care costs and production losses due to incapacity to work. In Europe, the mean annual cost per person with MS has been estimated at €23,000 for EDSS score 0.0–3.5, rising as disability increases to €46,000 for EDSS score 4.0–6.5, and €77,000 for EDSS score 7.0–9.5 (36).

Factors associated with TI include low volume of MS patients, non-specialty, and physicians’ ambiguity preferences (e.g., low tolerance to uncertainty in patient care, high aversion to financial ambiguity). Taken together, TI may be explained, at least in part, by (i) the aversion of neurologists to escalate treatment when the available options can have more serious side effects; (ii) the limited education (or experience) of neurologists regarding the risk profile of new DMTs, and (iii) participants’ preference to continue with a known medication profile vs. the unknown risks of a new agent. Other studies have found that TI was associated with lack of training and clinical uncertainty (5). Physicians with better coping strategies and more tolerance to ambiguity may be more likely to choose optimal treatments leading to better patients’ outcomes (37).

The prevailing significance of aversion to ambiguity in the financial over the health domain in the multivariate analysis may be related to either methodological differences when measuring each variable or suggest an underlying hardwired representation of aversion to ambiguity and TI that is easier elicited in the financial domain (18).

The results of DIScUTIR MS may not only be relevant for MS care but also be seen as the initial step to inform the design of a larger worldwide intervention, including physicians’ high aversion to ambiguity in the financial domain and low tolerance to uncertainty in patient care when assessing the use of new agents.

Our study has limitations that deserve comment. First, the study was conducted in Spain exclusively, thus limiting the generalizability of our results to other cultural contexts. Moreover, some participants may have responded based on their current local restrictions to prescribe specific DMTs. However, cognitive distortions and risk preferences have been identified in several studies of physicians’ decisions across the world and are thus probably not limited to a specific region or country (38). Second, the assessment of case scenarios may not fully capture decisions made in real clinical practice, even though specialists designed and recognized the scenarios as close to daily practice. In addition, participants may refer their MS patients to an MS outpatient clinic as part of a standard practice, which may have influenced our results. Third, considering the relatively low sample size, our findings should be viewed as exploratory. However, our results were consistent across several criteria of TI and adjusted models. Fourth, the concept and definition of TI applied to MS care is not widely disseminated and not yet generally accepted in MS care. Nevertheless, we used a practical definition of TI (absence of escalation in the face of a clinical relapse plus evidence of imaging activity), which is supported by consensus panels, as well as by MS studies and other areas showing improvements in clinical outcomes when escalating therapies (i.e., blood pressure and diabetes) (14, 39, 40).

Despite these limitations, our study is the first step in the understanding of how specific characteristics of physicians (i.e., high ambiguity aversion, low tolerance to uncertainty) directly influence therapeutic decisions in MS patients beyond demographic factors, medical expertise, practice setting, patients’ factors, or their treatment preferences. Using a novel approach that combines case-vignettes with the assessment of cognitive distortions through experiments from behavioral economics, we were able to expand our current understanding of decision-making under uncertainty in MS care.

Although MS experts have an expanded therapeutic arsenal compared to a decade ago, our study shows that nearly 7 out of 10 neurologists exhibited TI leading to suboptimal decisions. The results of DIScUTIR MS provide vital information to initiate discussions on behavioral strategies and incentives in order to ameliorate physicians’ inertia to escalate therapies leading to better outcomes and quality of life for MS patients (41). For example, training in risk management and decision-making, as well as, educational interventions are needed to overcome knowledge-to-action gaps (and reduce the TI) in MS care. This is relevant considering the lack of well-established MS guidelines concerning clinical scenarios under uncertainty or progression of disease and the limited understanding on how physicians’ preferences (e.g., aversion to ambiguity) have a global impact on medical and daily life decisions (42).

## Ethics Statement

All subjects gave consent (online) in accordance with the Declaration of Helsinki. The protocol was approved by the Research Ethics Board of St. Michael’s Hospital, University of Toronto, Canada.

## Author Contributions

GS: study concept and design, acquisition of data, analysis and interpretation of the data, and obtaining funding; AS: study concept and design, interpretation of the data, and critical revision of the manuscript for intellectual content; DP: interpretation of the data and critical revision of the manuscript for intellectual content; DS: study design, interpretation of the data, and critical revision of the manuscript for intellectual content; CR, JM, and PT: study concept and design, interpretation of the data, critical revision of the manuscript for intellectual content, and study supervision.

## Conflict of Interest Statement

PT and CR were funded by the Swiss National Science Foundation (PT: PP00P1_150739 and 00014_165884; CR: 105314_152891, CRSII3_141965, and 320030_143443). GS is supported by the Distinguished Clinicians Scientist Award from HSFC. AS, DP, DS, and JM have no disclosures.
