# Supplementary material for: Decision-making in Multiple Sclerosis: The Role of Aversion to Ambiguity for Therapeutic Inertia among Neurologists (DIScUTIR MS)
Source: Front Neurol. 2017 Mar 1;8:65. doi: 10.3389/fneur.2017.00065 (PMC5331032; doi:10.3389/fneur.2017.00065)
Supplement: Supplementary file 1 [file Data_Sheet_1.pdf]

APPENDIX

Table S1. Criteria of Therapeutic inertia and case-scenarios

Criteria	Clinical + Radiological	EMA criteria	Composite modified Rio ≥2 or MS progression
**Case-scenario number**			
	7	4	4
	8	8	7
	13	14	13
	14	15	14
			15
**Definition**	Clinical relapse + at least 1 Gad enhancing lesion	1 relapse last year + ≥ 9 new T2 OR ≥ 1 Gad T1	MRI criterion: 1 if >5 new T2 lesions<br>Clinical criterion:<br>1 if 1 relapse<br>2 if ≥ 2 relapses over the first year or EDSS ≥1 from baseline
**Overlap with other measures**	**NO**	Herding	**NO**

Table S2. Variables associated with therapeutic inertia: full logistic regression models

	Clinical + Radiological	EMA	Modified Rio or progression
	OR; 95% CI		
Ambiguity aversion- (Financial domain)	8.6 (1.01-73.32)	7.16; 1.36-37.63	3.91; 0.63-23.96
Ambiguity aversion- (Health domain)	0.75 (0.17-3.34)	0.16; 0.30-0.92	1.10; 0.26-4.56
Age	0.93; 0.81-1.07	1.07; 0.84-1.36	0.95; 0.85-1.07
Gender, male	0.54; 0.18-1.67	0.85; 0.28-2.51	0.52; 0.17-1.56
Years in practice	1.02; 0.89-1.17	0.90; 0.69-1.17	1.01; 0.91-1.13
Authorship	1.18; 0.24-5.69	2.81; 0.73-10.74	1.37; 0.28-6.5
Patients seen/week	0.96; 0.93-1.00	0.96; 0.91-1.01	0.97; 0.93-1.00
Setting, academic	1.48; 0.41-5.28	0.44; 0.12-1.60	1.25; 0.36-4.31
Attendance ECTRIMS 2015	1.38; 0.44-4.30	1.60; 0.54-4.77	1.26; 0.41-3.85
SOEP	0.74; 0.25-2.18	1.26; 0.43-3.70	0.87; 0.30-2.5
Low tolerance to uncertainty	3.9; 1.22-12.45	0.65; 0.21-1.93	4.27; 1.36-13.4
Herding experiment	0.37; 0.06-2.08	0.36; 0.09-1.37	0.34; 0.062-1.85
Risk Aversion	0.70; 0.18-2.68	2.35; 0.62-8.86	0.83; 0.22-3.11
Overconfidence	1.21; 0.30-4.89	1.20; 0.34-4.22	1.35; 0.34-5.37

Figure S1. Representation of participants in DIScUTIR MS

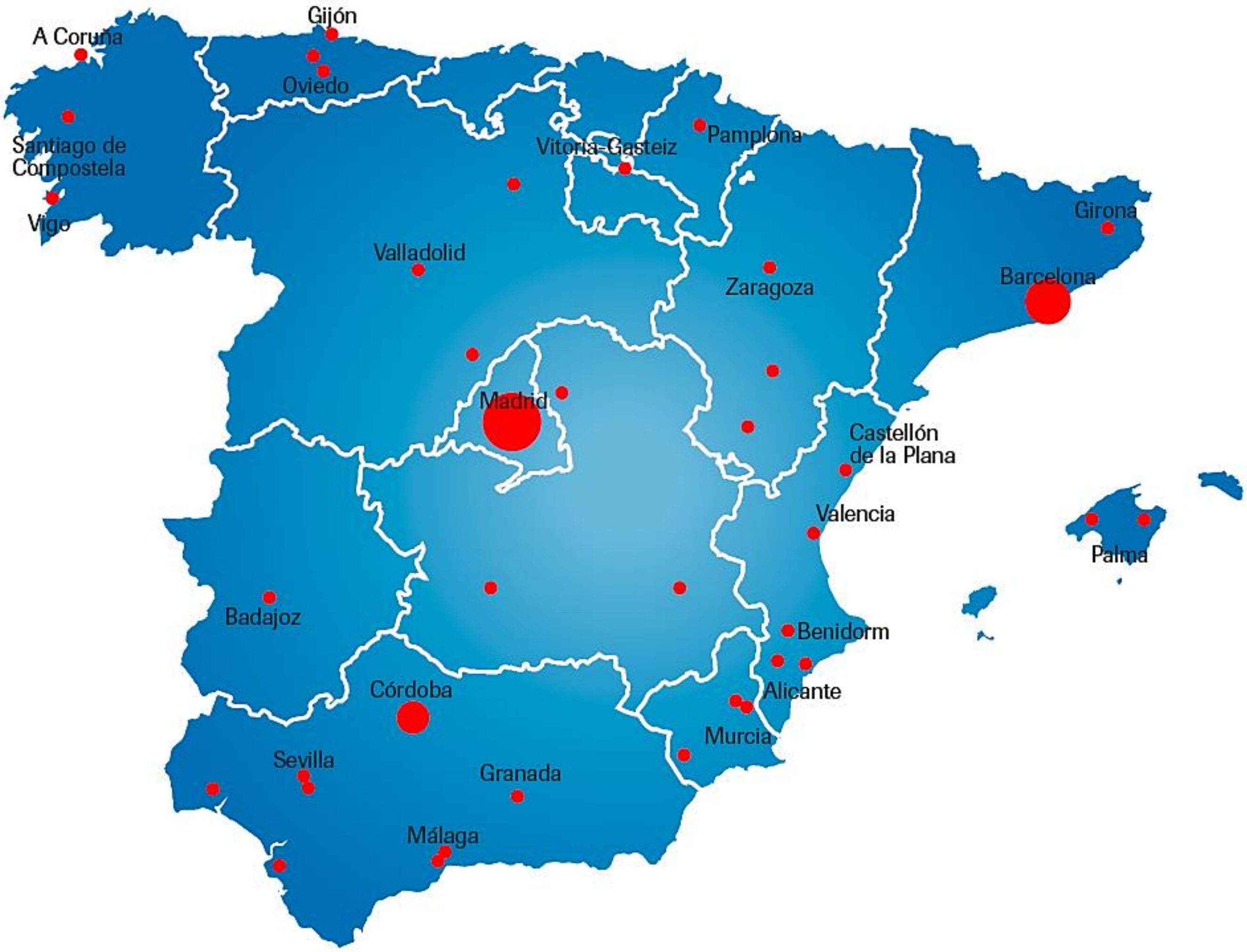

Participants from all territories in Spain were represented. Dot sizes represent the number of participants in each district.

Figure S2. Preference for the known probability (50/50) option for each the nine scenarios with unknown probability (ambiguity aversion)

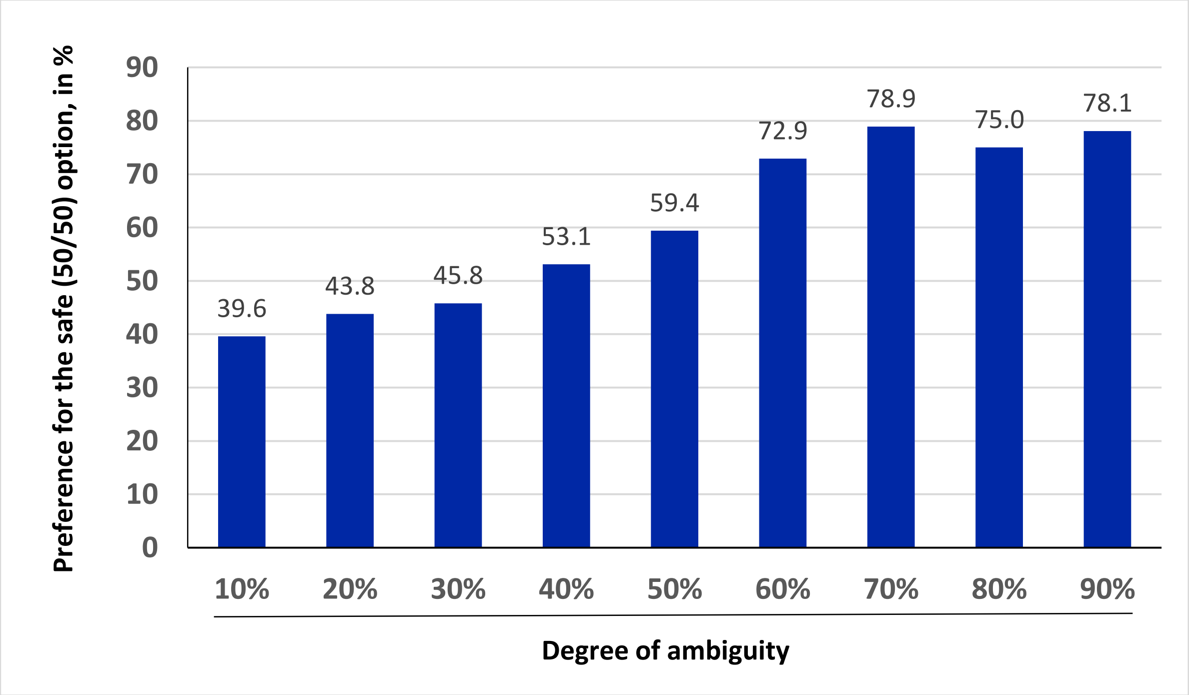

Note the increasing number of participants choosing the known probability option (aversion to ambiguity) when the unknown probability increases.

Figure S3. Number of times that participant choose the known probability (50/50) option over the ambiguous option

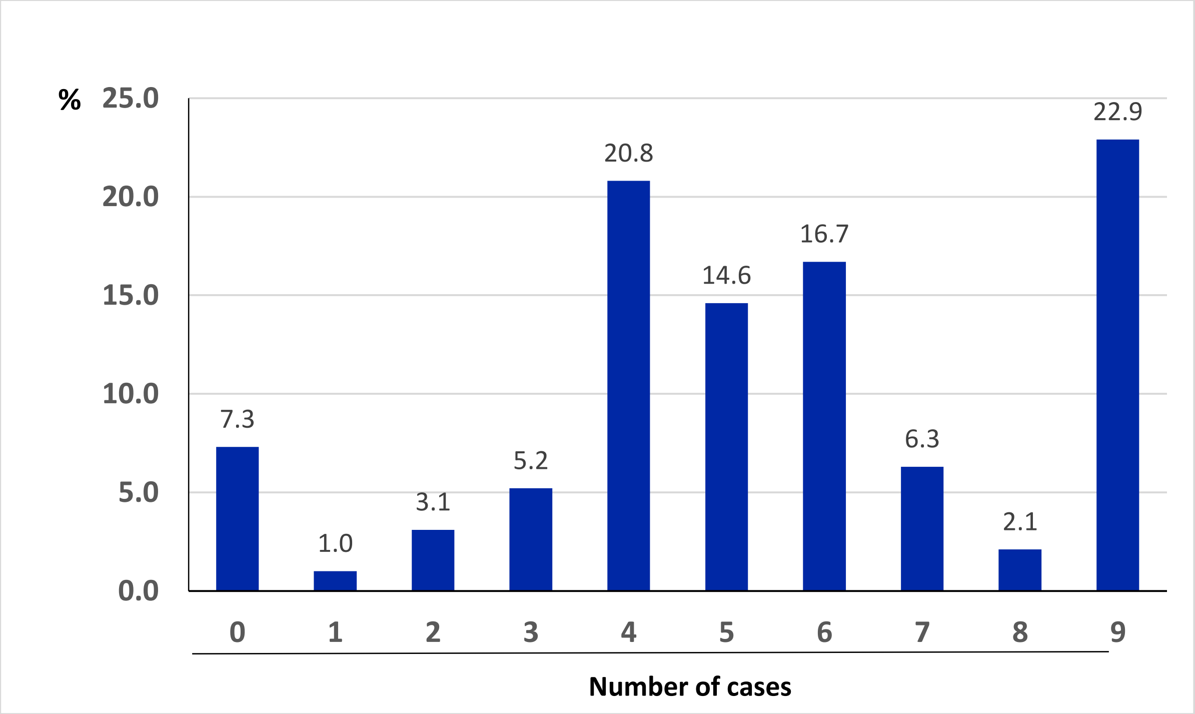

Note: This figure represents the number of scenarios that participants choose the known probability option (50/50) over the ambiguous (unknown probability) option. For example, 20.8% of participants selected the 50/50 option in 4 scenarios, 16.7% selected the 50/50option in 6 scenarios. Overall, 22.9% of participants selected the known probability (50/50) option in all nine scenarios suggestive of complete aversion to ambiguity.
